# Investigating the impact of breast positioning control on physical treatment parameters in multi-catheter breast brachytherapy

**DOI:** 10.1007/s00066-023-02127-0

**Published:** 2023-08-17

**Authors:** Andre Karius, Vratislav Strnad, Michael Lotter, Stephan Kreppner, Rainer Fietkau, Christoph Bert

**Affiliations:** 1 Department of Radiation Oncology, Universitätsklinikum Erlangen, Friedrich-Alexander-Universität Erlangen-Nürnberg, Universitätsstr. 27, 91054 Erlangen, Germany; 2 Comprehensive Cancer Center Erlangen-EMN (CCC ER-EMN), Erlangen, Germany

**Keywords:** Adaptive brachytherapy, Implant stability, Treatment quality, Computed tomography, Treatment re-planning

## Abstract

**Purpose:**

To assess the effects of a workflow for reproducible patient and breast positioning on implant stability during high-dose-rate multi-catheter breast brachytherapy.

**Methods:**

Thirty patients were treated with our new positioning control workflow. Implant stability was evaluated based on a comparison of planning-CTs to control-CTs acquired halfway through the treatment. To assess geometric stability, button–button distance variations as well as Euclidean dwell position deviations were evaluated. The latter were also quantified within various separated regions within the breast to investigate the location-dependency of implant alterations. Furthermore, dosimetric variations to target volume and organs at risk (ribs, skin) as well as isodose volume changes were analyzed. Results were compared to a previously treated cohort of 100 patients.

**Results:**

With the introduced workflow, the patient fraction affected by button–button distance variations > 5 mm and by dwell position deviations > 7 mm were reduced from 37% to 10% and from 30% to 6.6%, respectively. Implant stability improved the most in the lateral to medial breast regions. Only small stability enhancements were observed regarding target volume dosimetry, but the stability of organ at risk exposure became substantially higher. D_0.2ccm_ skin dose variations > 12.4% and D_0.1ccm_ rib dose variations > 6.7% were reduced from 11% to 0% and from 16% to 3.3% of all patients, respectively.

**Conclusion:**

Breast positioning control improved geometric and dosimetric implant stability for individual patients, and thus enhanced physical plan validity in these cases.

**Supplementary Information:**

The online version of this article (10.1007/s00066-023-02127-0) contains supplementary material, which is available to authorized users.

## Introduction

Multi-catheter brachytherapy as accelerated partial breast irradiation (APBI) has been well established as standard procedure for adjuvant radiotherapy of early-stage breast cancer following breast-conserving surgery [1]. Pioneering studies have shown the non-inferiority regarding local control compared to external beam radiation therapy (EBRT) [2, 3]. Simultaneously, improved toxicity and very good results regarding cosmetic outcomes and quality of life are achievable [4, 5].

Despite the established and high clinical value of breast brachytherapy, recent studies described the occurrence of geometric implant instabilities during the treatment course for individual patients [6, 7]. These also affected target volume and organ at risk (OAR) dosimetry, which rendered treatment re-planning necessary. The underlying causes of implant instabilities as particularly arm position differences, varying breast muscle tension, and breast tissue compressions were described previously [6].

As consequence of these findings, we introduced a breast positioning control during brachytherapy as routine procedure. This aimed to standardize both patient and breast position during all irradiation fractions individually adapted to each patient. So far, 30 patients were treated with this workflow. For the present work, an assessment of the corresponding implant stability compared to a formerly treated cohort of 100 consecutive patients was performed. Hereinafter the stability enhancements achieved with our new workflow are reported.

## Materials and methods

### General workflow

All patients received multi-catheter brachytherapy as APBI following breast-conserving closed-cavity surgery for a duration of 4–5 days with a fractionation scheme of 9 × 3.8 Gy or 7 × 4.3 Gy. For this procedure, flexible plastic catheters (type 6F, Elekta, Veenendaal, Netherlands) were implanted into the breast under radiography control following GEC-ESTRO guidelines [8]. Plastic buttons were applied at the patient skin to prevent slippage.

Treatment planning was conducted within the software Oncentra Brachy (Nucletron, Veenendaal, Netherlands) based on a planning computed tomography scan (P-CT) acquired in expiration with 2 mm slice thickness and 0.3–0.6 mm pixel size. The planning target volume (PTV), OARs (ribs, skin considered as 5 mm shell underneath the body surface), and breast were contoured following ESTRO guidelines [8, 9]. Catheter paths in-situ were manually reconstructed, after which dwell positions and times of an ^192^Ir afterloader source were manually defined. For the latter, following dose constraints to PTV and OARs were considered in accordance with ESTRO guidelines [8]: i) D_0.2ccm_ and D_1ccm_ of the skin < 100% and < 90% of the prescribed dose, respectively; ii) D_0.1ccm_ and D_1ccm_ of the ribs < 90% and < 80%; iii) V_100_ and D_90_ of the PTV ≥ 90% and ≥ 100%. D_xccm_ and D_x_ refer to the dose the most exposed x ccm or x% of the structure receive, respectively, and V_x_ to a structure’s volume receiving at least x% of the prescribed dose. Furthermore, a dose non-uniformity ratio [8] (DNR) ≤ 0.35 and conformity index [8] (COIN) ≥ 0.65 is aimed at.

After the fourth irradiation fraction, a control-CT (C-CT) acquisition identical to the P‑CT was performed as routine procedure. On this C‑CT, catheters were manually reconstructed and dwell positions and times from the treatment plan were transferred accordingly. This served to identify patients requiring treatment re-planning by following a decision-tree methodology published previously [6].

Note that in our previous treatment workflow, the technicians visually checked the correct location of the distal buttons (i.e., a position as close as possible to the skin) prior to each fraction, the P‑CT, and the C‑CT as only quality control (QC) procedure. This referred to a sole visual assessment without any markings comparable to the descriptions of the “Breast positioning control” section or similar being used. In case of displacements, the buttons were manually shifted by eye back towards the skin. No further QC measures prior to each fraction, the P‑CT, or C‑CT were taken. In particular, no special attention was paid to position the patients in exactly the same way, especially with respect to arm positions, as positioning for the P‑CT was conducted. In this work, we considered 100 patients treated with this previous workflow as control group to the patients treated after introducing breast positioning control.

### Breast positioning control

**Fig. 1:**
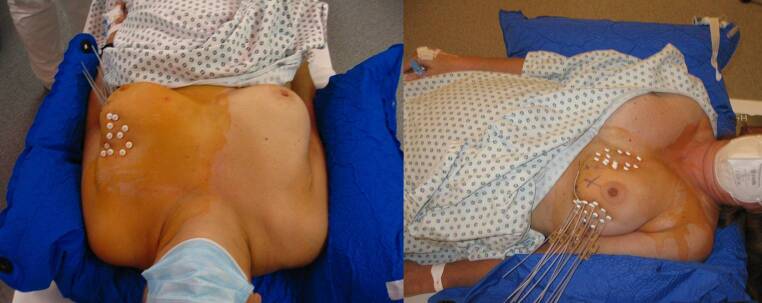
Shown is the positioning of two patients within an individually adapted vacuum cushion, as intended by the breast positioning control workflow to increase implant stability by positional stability

Karius et al. [6] recently identified particularly arm position differences, varying breast muscle tension, and breast tissue compressions as major causes for implant alterations during breast brachytherapy. As consequence, we started a breast positioning control workflow throughout the treatment course as routine procedure. This means all patients were positioned within a vacuum cushion individually adapted to the patient body right before the P‑CT (Fig. 1), with the arms being flat-lying on each body site and the head placed on a flat cushion. As in the past, care was taken that the distal buttons were placed as close as possible to the skin. As further component of the new breast positioning control workflow, we additionally checked the appropriate positioning of the proximal buttons which were intended to match corresponding markings on the catheters. These markings were manually created with a black pencil and referred to the corresponding button positions at the time of P‑CT. This breast positioning control was conducted prior to each irradiation fraction, the P‑CT, and the C‑CT for the treatment of 30 patients so far. No further adaptions or changes with respect to our previous treatment workflow described in the “General workflow” section were conducted. Note that, since our new workflow was completely established as our new treatment QC standard starting from its clinical implementation, IRB ethics approval was neither obtained nor became necessary for the present work. In the following, the corresponding implant stability observed for the patients treated with and without breast positioning control was compared to each other.

### Geometric implant stability

To evaluate implant stability, we focused on an assessment of button–button distance variations and dwell position deviations. For the first, we measured for each catheter the distance between both associated buttons along the catheter path and calculated the corresponding differences between P‑CT and C‑CT [6]. This was of pertinence, since respective length deviations might be indicators for breast deformations leading to dosimetric variations regarding PTV and skin. Furthermore, measuring these distances also allows validation of treatment compliance with our implemented breast positioning control.

Considering dwell position deviations provided a measure for geometric implant stability with immediate dosimetric impact. For this reason, we measured for each dwell position of each implanted catheter the mean Euclidean distance to all individual dwell positions of all other implanted catheters. Calculations were done automated via an in-house Python script by reading out the corresponding RT-Plan files created in Oncentra Brachy. This procedure was performed on both P‑CT and C‑CT and the corresponding differences obtained for each dwell position were calculated. To analyze the location-dependency of dwell position deviations, each P‑CT breast contour was separated into 125 regions, i.e. into 5 divisions parallel and orthogonal to the chest wall and into 5 divisions along the cranial–caudal direction. For each region and all dwell positions located within it, the mean Euclidean distance difference was separately evaluated as mentioned above. To visualize the findings as colormap, the results for all patients (separated by cohort) and both chest sides were projected into the illustration of an axial and coronal breast contour.

### Dosimetric implant stability

**Fig. 2:**
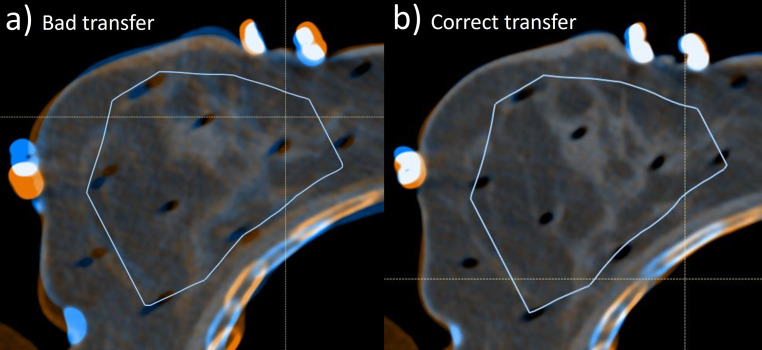
Shown is an example for evaluating the correct placement of the target volume (*blue solid line*) that was created on the planning-CT (P-CT; *blue CT image*) within the control-CT (C-CT; *orange CT image*). In this case, using rigid registration (**a**) resulted in insufficient transfer and registration quality, since both catheter paths as well as breast tissue signature clearly partly did not overlap, as illustrated by the different colors. Using the deformable image registration (**b**), however, enabled a correct target volume transfer. In this case, both catheter paths and tissue signature overlapped almost perfectly, since within the breast region almost no separated colored parts referring to the individual CT scans were observed anymore

To evaluate dosimetric stability, skin and ribs were re-contoured on the C‑CT. Furthermore, the PTV was transferred from the P‑CT into the C‑CT using either the rigid image registration of Oncentra Brachy or the deformable image registration of the software RayStation (RaySearch, Stockholm, Sweden), depending on what was required for a correct structure placement within the C‑CT. This correct placement was visually checked by evaluating the correct locations of the contour margins with respect to the individual catheter paths and the breast tissue signature as shown in Fig. 2. Furthermore, registration quality was classified by determining the registration quality score suggested by the AAPM TG 132 report [10]. All dose metrics mentioned in the “General workflow” section were again evaluated and compared to the treatment plan. In addition, the volumes enclosed by the 100% and 150% isodose surfaces were determined on both P‑CT and C‑CT. Their variations indicated dose volume deviations originating solely from geometric implant alterations without relation to contours.

To determine the necessity for treatment adaption for each patient, the decision-tree published by Karius et al. [6] was applied and is visualized as Supplementary Material. This essentially considers skin and PTV dosimetry to make final decisions about whether patients are candidates for re-planning. The latter was particularly the case if the skin dose D_0.2ccm_ and D_1ccm_ exceeded the constraints given in the “General workflow” section and simultaneously revealed P‑CT–C-CT changes ≥ 10% of the prescribed dose. Furthermore, this was also the case if the PTV revealed a D_90_ < 100% and V_100_ < 90% with simultaneous P‑CT–C-CT variations ≥ 3% of the prescribed dose. The number of patients requiring re-planning was determined for both examined patient cohorts.

### Statistical analysis

In this work, the implant stability of patients undergoing breast positioning control was compared to patients treated prior to its implementation. For all examined geometry and dose metrics, statistical significance of occurring differences was determined by applying chi-square distribution tests at significance level 5%.

## Results

### Geometric implant stability

**Fig. 3:**
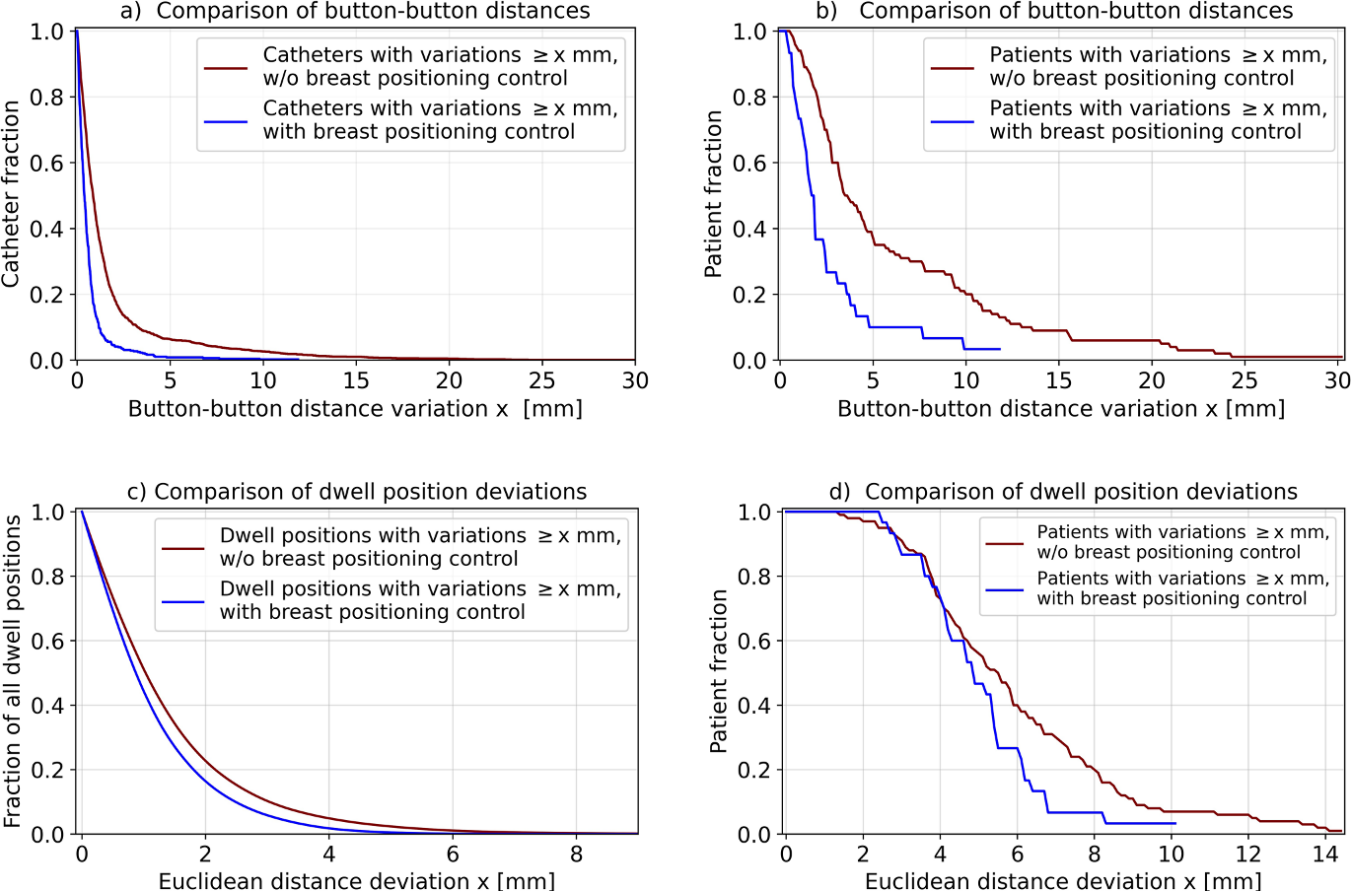
Shown in Kaplan–Meier style are the relative fractions of implanted catheters (**a**) and patients (**b**) affected by at least one button–button distance variation with an amount larger than the corresponding abscissa value for the patient cohorts treated with and without the breast positioning control workflow. The fractions of dwell positions (**c**) and patients (**d**) affected by respective Euclidean dwell position deviations are illustrated as well

Breast positioning control resulted in substantial improvements of geometric implant stability. With this workflow, the mean amount of button–button distance variations was 0.6 ± 1.0 mm, compared to 1.6 ± 2.7 mm obtained for the previous cohort (Fig. 3a). Only 0.4% and 0.4% of the 485 catheters implanted in the 30 patients showed a length shortening and extension > 5 mm (referring to one button size), respectively, whereas this was the case for 2.7% and 3.5% of the 1758 catheters implanted in the 100 patients treated beforehand. Distance deviations with an amount > 1 mm and > 2 mm were measured for 13.6% and 4.5% (with positioning control) as well as for 43.6% and 18.7% (without positioning control) of the implanted catheters. The number of patients affected by large deviations could be significantly reduced as well (Fig. 3b). When treated with the implemented workflow, only 36.7% and 10% of patients revealed at least one distance variation with an amount > 2 mm and > 5 mm, respectively, compared to 80% and 37% of the previous patients. No patient treated with the workflow showed variations > 12 mm, whereas this was observed for 13% of the previous patients. Thus, button-button distance variations were, applying the chi-square distribution tests mentioned in the “Statistical Analysis” section, significantly reduced in terms of both the number of affected catheters (*p* < 0.001) and patients (*p* < 0.001).

**Fig. 4:**
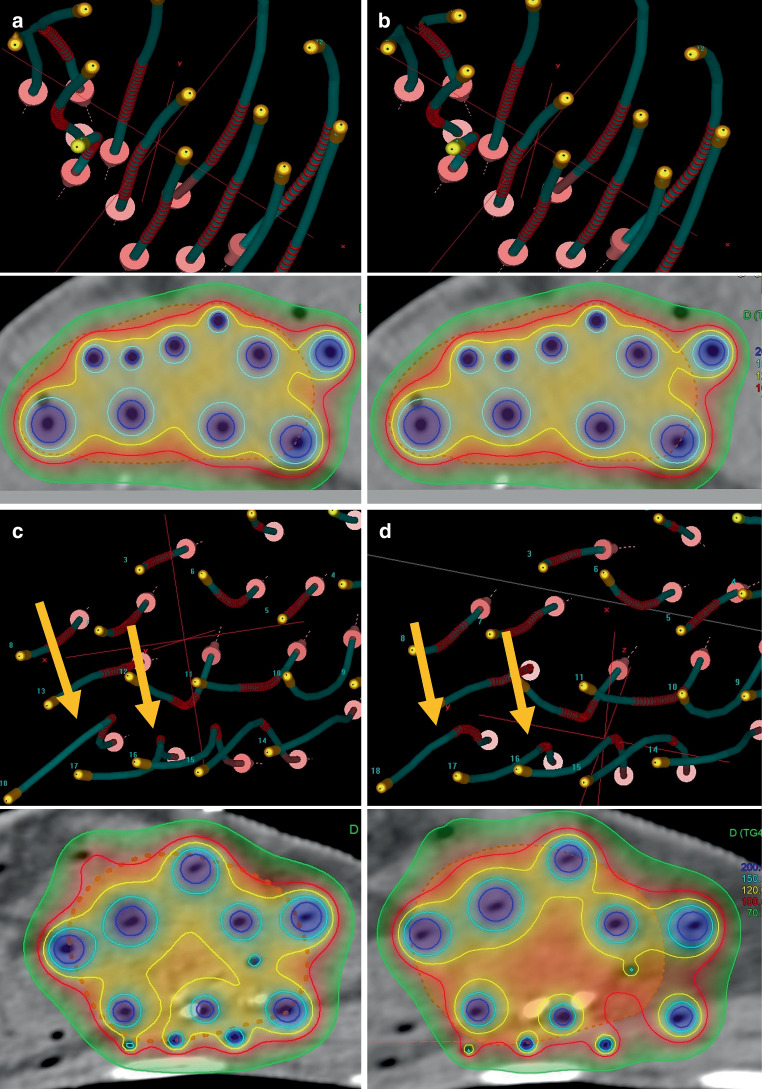
**a** and **b** show the planning-CT and control-CT situation, respectively, of a patient with very small geometric changes (dwell position deviations < 1 mm) treated with breast positioning control; **c** and **d** show the planning-CT and control-CT situation, respectively, of a previously treated patient with large dwell position deviations (up to 8 mm). In this case, catheters that clearly changed their paths within the breast are exemplarily marked with *orange arrows*. These changes resulted in an underdosage of some target volume parts. Catheter paths (*blue*) are in each case shown together with the source dwell positions (*red*) in the upper sections of each subfigure. The resulting dose distribution is visualized in the lower sections. The target volume is marked *orange* (*dashed*) in each case. The 70% (*green*), 100% (*red*), 120% (*yellow*), 150% (*cyan*), and 200% (*dark blue*) isodose lines are displayed

Regarding the dwell position deviations, breast positioning control led to stability enhancement as well. The mean amount of corresponding deviations was 1.1 ± 1.0 mm when applying the new workflow and 1.4 ± 1.4 mm for the previous patients (Fig. 3c). As example, 16.4% and 1.7% of the dwell positions defined for the patients receiving breast positioning control showed deviations > 2 mm and > 4 mm, respectively, compared to 22.7% and 4.9% obtained for the patients treated before. In particular, Euclidean deviations were significantly reduced regarding both the number of affected dwell positions (*p* < 0.001) and of affected patients (*p* < 0.001). Although corresponding deviations with an amount > 5 mm were still observed in 46.7% of the patients treated with breast positioning control (compared to 56% of the patients treated without), only 6.6% of the patients showed a deviation > 7 mm (Fig. 3d). For the previous cohort, such variations were found for 30% of all patients and deviations up to 14.5 mm occurred. To visualize examples of the observed implant alterations, Fig. 4 shows the geometric and dosimetric variations for a patient with very small and very large implant variations, respectively. Furthermore, corresponding changes for the patients showing the maximum implant alterations of both cohorts are provided as Supplementary Material.

**Fig. 5:**
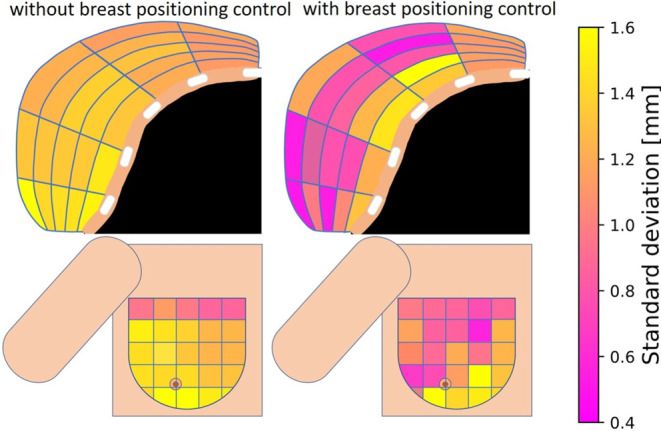
Provided as colormaps are the standard deviations of the Euclidean dwell position deviations observed in the various breast segments, projected into an illustrative axial and coronal breast view for each cohort

Breast positioning control improved implant stability particularly in the lateral to medial parts of the breast. As illustrated in the colormaps (Fig. 5), the lateral breast regions close to the patient arm showed respective standard deviations of the dwell position variations of 0.5–1.2 mm for patients treated with positioning control compared to 1.2–1.5 mm obtained for the previous cohort. However, almost no changes in dwell position deviations were observed in the segments adjacent to chest wall. Here, standard deviations of 1.1–1.5 mm were obtained for both patient cohorts. The coronal breast projections showed in each case only small standard deviations in the most cranial segments of 0.6–1.1 mm and 0.9–1.1 mm for patients with and without positioning control, respectively. The most caudal regions corresponded to the segments most affected by dwell position deviations in both cases.

### Dosimetric implant stability

**Fig. 6:**
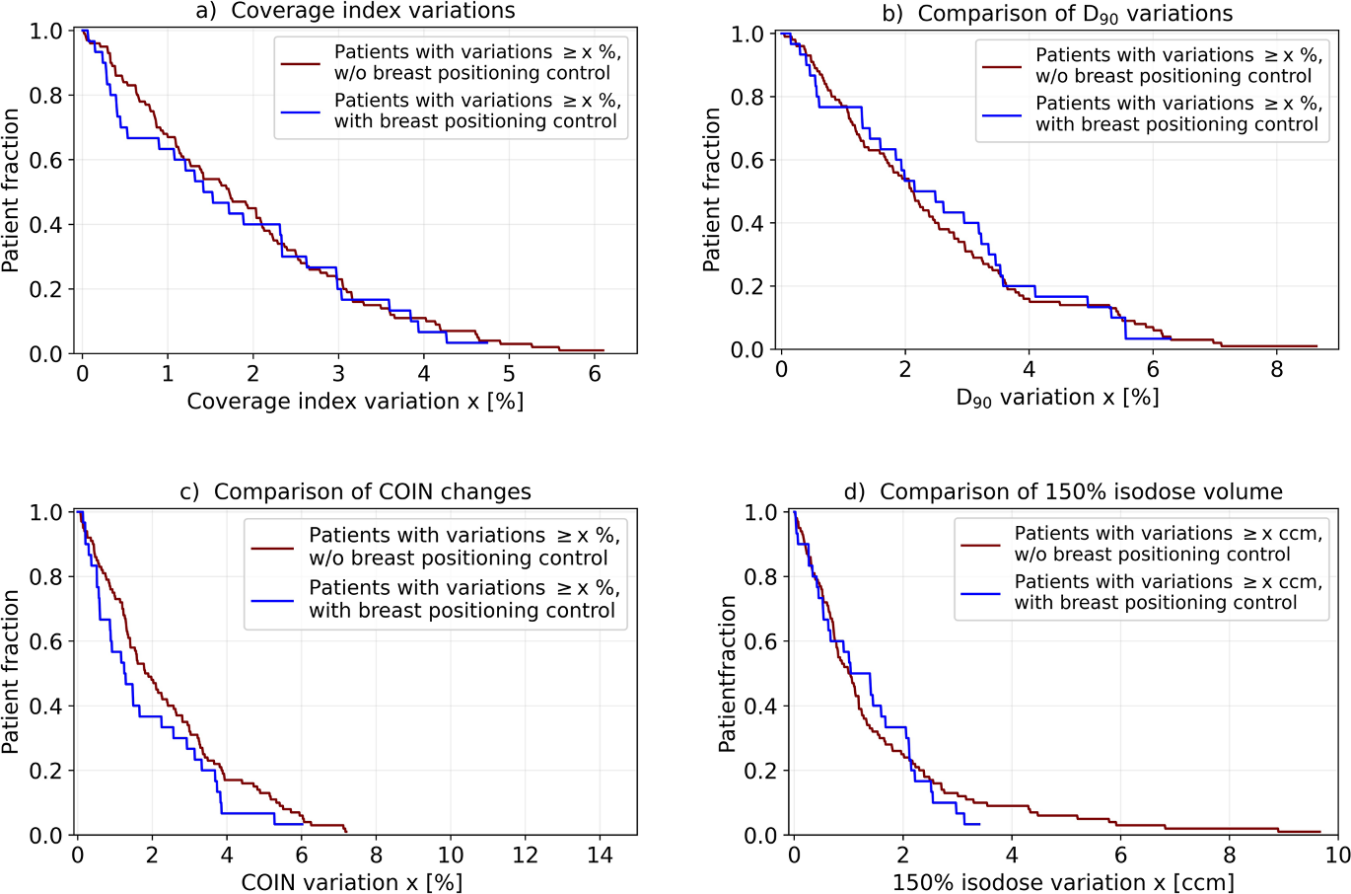
Shown in Kaplan–Meier style are the relative fractions of all patients that were affected by coverage index (**a**), D_90_ (**b**), COIN (**c**), and 150% isodose volume (**d**) variations with an amount larger than the corresponding abscissa value. Results are provided for both patient cohorts treated with and without the breast positioning control workflow

Geometric implant alterations resulted for both patient cohorts in dosimetric instabilities. However, regarding the PTV, dosimetric variations were almost identical for patients treated with and without breast positioning control. The mean amount of coverage index CI (i.e. V_100_) and D_90_ changes was 1.8 ± 1.4% and 2.5 ± 1.8% for patients receiving the new workflow, respectively, and 1.9 ± 1.4% and 2.5 ± 1.8% for the previous patients (Fig. 6a, b). Nevertheless, we also observed a trend for eliminating larger coverage index and D_90_ variations, since only one patient treated with positioning control showed variations > 4.3% and > 5.6%, respectively. For the previous cohort, 7% and 9% of the patients were affected by such coverage index (up to 6.1%) and D_90_ (up to 8.6%) changes. Slight improvements were also observed regarding the COIN (Fig. 6c). For patients treated with and without positioning control, the mean amount of COIN changes was 1.3 ± 2.1% and 2.4 ± 1.9%, respectively. One patient receiving the new workflow showed variations > 5.3%, whereas this was observed for 11% of the previous patients.

The 100% isodose volumes changed on average by 1.0 ± 1.6 ccm and 0.8 ± 1.3 ccm for patients treated with and without breast positioning control, respectively. However, the workflow substantially improved the stability of high-dose areas, since the number of patients with large corresponding variations was markedly reduced (Fig. 6d). For instance, no patient treated with the new workflow showed respective changes of the 150% isodose volumes > 3.4ccm, whereas this was the case for 10% of the patients examined before. The latter revealed a maximum variation of 9.7 ccm. As consequence, only one patient receiving positioning control showed absolute DNR changes > 2.4% compared to 13% of the patients treated prior (absolute change of 10.2% at maximum).

**Fig. 7:**
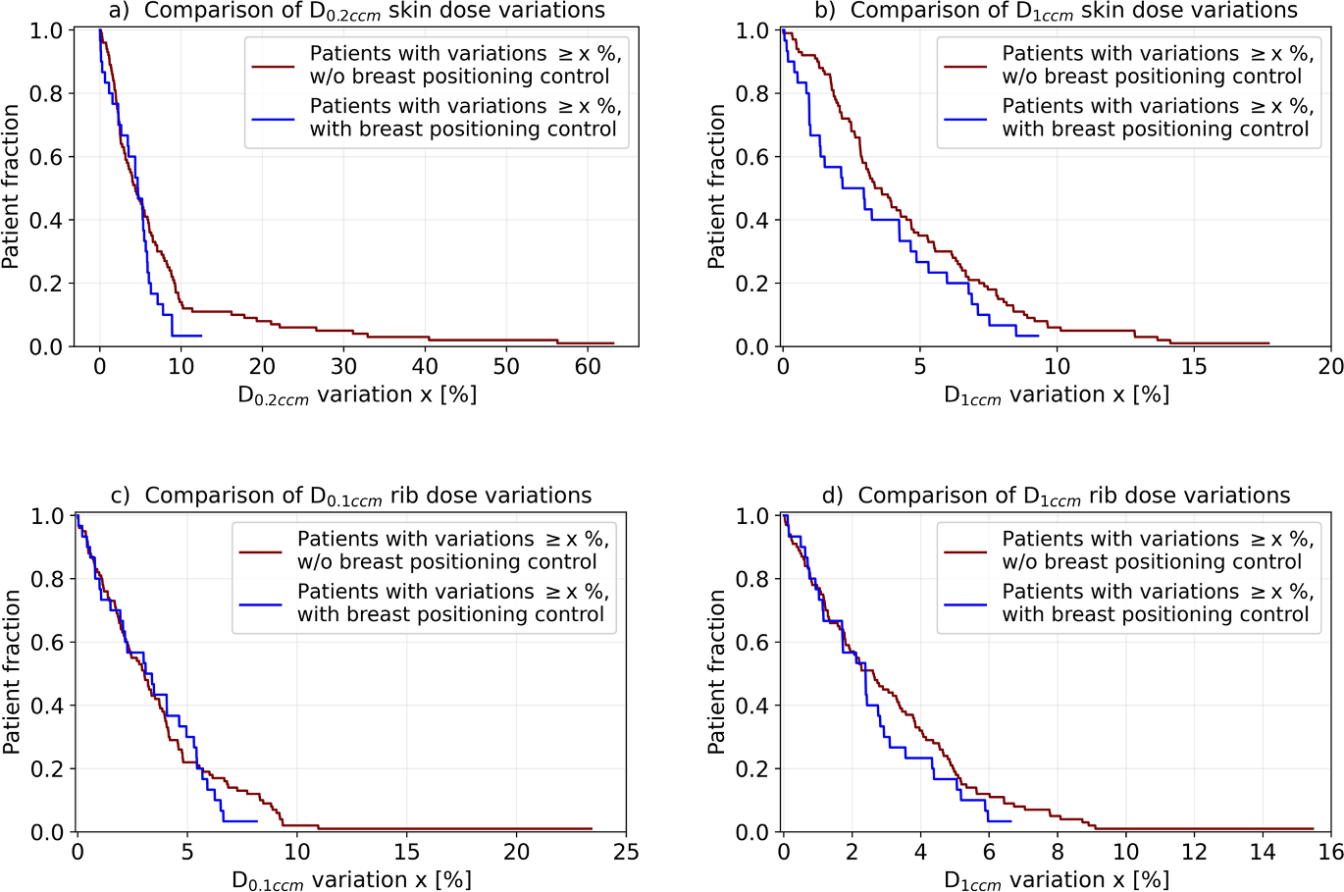
Shown in Kaplan–Meier style are the relative fractions of all patients that were affected by absolute D_0.2_ _ccm_ (**a**) and D_1_ _ccm_ (**b**) skin dose changes as well as by absolute D_0.1_ _ccm_ (**c**) and D_1_ _ccm_ (**d**) rib dose changes with an amount larger than the corresponding abscissa value. Results are provided for both patient cohorts treated with and without the breast positioning control workflow

The greatest benefit of breast positioning control was found regarding the exposure to OARs. For instance, no patient treated with the workflow showed absolute D_0.2ccm_ and D_1ccm_ skin dose changes with an amount  > 12.4% and > 9.3% of the prescribed dose, respectively, whereas these were observed for 11% and 8% of the patients treated before (Fig. 7a, b). For the latter, the maximum D_0.2ccm_ and D_1ccm_ changes amounted 63.1% and 17.7% of the prescribed dose, respectively. Note, that we are reporting the skin dose changes as fraction of the prescribed dose and therefore describe absolute and not relative dosimetric deviations at this point. Hence, regarding both dose metrics, a substantial reduction of large variations and significantly (*p* ≤ 0.001 in both cases) improved skin exposure stability was achieved. The mean amount of D_0.2ccm_ variations was 4.4 ± 3.0% and 7.4 ± 10.3%, and the mean amount of D_1ccm_ variations 3.3 ± 2.8% and 4.6 ± 3.4% for patients treated with and without positioning control, respectively. The ESTRO D_0.2ccm_ and D_1ccm_ skin dose constraints [8] were missed by no patient receiving the new workflow on the C‑CT, but by 12 and 4 patients treated without it.

Regarding the rib exposure, breast positioning control significantly improved D_0.1ccm_ (*p* < 0.001) and D_1ccm_ (*p* < 0.001) stability as well (Fig. 7c, d). Although only small changes of the mean variation amount (from 3.7 ± 3.4% to 3.4 ± 2.3% of the prescribed dose for D_0.1ccm_ and from 3.1 ± 2.6% to 2.6 ± 1.8% of the prescribed dose for D_1ccm_) were observed after introducing the new workflow, larger dose instabilities could be avoided. For instance, D_1ccm_ variations of > 4% of the prescribed dose were found for 21.4% of the patients receiving breast positioning control, compared to 32% of the previous patients. Furthermore, only 3.3% of the patients (referring to one patient) showed D_0.1ccm_ variations > 6.7% of the prescribed dose, whereas 16% of the patients treated before did.

### Requirements for treatment re-planning

In summary, with breast positioning control an improved implant stability was achieved. In particular, large variations regarding high-dose areas and OAR exposure could be substantially reduced or even avoided. Dosimetric stability was also of fundamental importance regarding the decision-tree [6] applied to identify patients requiring treatment adaption. In this respect and as shown in the Supplementary Material, 14% and 1% of the patients treated without breast positioning control required re-consideration due to skin dose variations and target coverage loss, respectively. In contrast, for patients receiving breast positioning control, no individual was identified for re-planning. The effort for the assessment of the need for re-planning was reduced as well, since 67% of the patients (compared to only 55% of the previous cohort) did not require a detailed evaluation of dosimetric implant stability because they showed a high geometric implant stability (see Supplementary Material) in first line. The new workflow therefore effectively helped to avoid additional effort during the brachytherapy course, to enable smooth workflows, and to ensure high treatment quality.

## Discussion

In the present work, we introduced and established a breast positioning control workflow for daily routine, with reproducible patient positioning during the treatment course individually adapted to each patient, and evaluated the corresponding implant stability for 30 patients. Results were compared to a patient cohort treated previously. We observed a reduction of larger geometric and dosimetric alterations, and thus breast positioning control improved physical plan stability particularly for individual patients. No single patient was identified for re-planning when receiving the new workflow. As consequence, associated effort for physicians and physicists was eliminated, and patient safety as well as high treatment quality were ensured. Of course, the creation of vacuum cushion and catheter markings requires additional time at first (< 5 min for vacuum cushion, < 2 min for catheter marking), which, however, is considered substantially less than the time needed for a re-planning procedure.

Effects of breast positioning control on clinical outcomes such as local control and side effects will get accessible in the next few years for the first time. For this reason, our analysis only relates to physical implant stability, which we were able to improve. We believe that a large number of patients are required to clearly outline and validate actual clinical benefits, since breast brachytherapy represents even without positioning control an excellent treatment modality with local control rates of up to 98% [2, 3] and reasonable toxicity [4, 11]. Thus, for the majority of patients, multi-catheter breast brachytherapy is a reliable and stable treatment modality also without the introduction of our new workflow. Nevertheless, based on our results and since we managed to reduce and avoid the occurrences of comparatively larger geometric and dosimetric variations, we have the opinion that this workflow can be of high importance for individual patients. In this respect, particularly the improved stability of high-dose areas known to impact the formation of side effects such as fibrosis [12] has to be considered. Furthermore, the loss of dose conformity (measured via the COIN) and dosimetric variations to OARs could be substantially reduced. Breast positioning control thus appears of high relevance particularly for patients already exhausting the ESTRO dose constraints [8], which have to be treated very sensible with respect to potential dosimetric alterations. Considering these aspects, breast positioning control offers a possibility for treatment QC. Moreover, in our opinion, a treatment should always be delivered as closely as possible to the respective treatment plan, and our workflow forms a small mosaic component helping to achieve this goal.

Despite breast positioning control and although we managed to reduce the button–button distances primarily by our catheter markings in general, some button–button distance variations even of > 5 mm (referring to one button size), which were found to correlate significantly with skin dose variations [6], were still observed. However, considering the catheter markings as essential component of our new workflow, especially the maximum distance deviations of up to 10 mm should have not occurred in principle. At this point, it remains unclear whether these variations were due to a human mistake in button positioning, non-compliance with the workflow, or due to tissue effects such as breast swelling. In any case, our observations disclose the absolute necessity of a complete and seamless positioning control prior to each individual irradiation fraction. The change to different catheter systems ensuring a constant button–button distance during the treatment course is considered supportive in this respect.

 Furthermore, breast positioning control improved implant stability mainly in the lateral to medial breast regions (Fig. 5). This is compliant to recent findings [6], which identified differences in arm positions and breast muscle tensions as one major factor for implant variations. Varying breast muscle tensions can especially impact the paths of catheters implanted close to the chest wall (i.e., the catheters of the lowest implant row), as described previously [6]. In this respect, the catheter locations within the breast are considered most relevant for the occurrence of corresponding changes, since, particularly, no correlations of breast size and geometric variations seem to exist [6]. Since our workflow includes particularly reproducible arm fixations, these factors were controlled and corresponding variations therefore reduced. One could argue that such improvements by reproducible positioning are expected, but nevertheless we provide for the first time ever a respective quantification of the associated stability enhancements.

Previous studies on implant stability during multi-catheter breast brachytherapy [6, 7] have already described in detail the magnitude and causes of implant variations occurring during the APBI course. However, as mentioned, the present work now quantifies for the first time the physical plan stability improvements achievable under consideration of fixed patient positioning. Our new workflow ensures, by the individual adaption of the vacuum cushion to the patient as described in the “Breast positioning control” section, a reproducible positioning during each irradiation fraction individually adapted to each patient. This procedure revealed to be sufficient for improving treatment plan stability over time for individual patients. However, factors such as breast tissue alterations or variations in the patient breathing phase, which may also lead to implant changes as described previously [6], are until now not accounted for by our new workflow.

Despite excellent clinical outcomes [2, 13], breast brachytherapy is still an evolving modality. In particular modern imaging techniques [14, 15] for image-guided implantations and treatment QC as well as novel fractionation concepts such as very accelerated partial breast irradiation [16, 17] are subject to current research. Our workflow complements these advancements and helps to improve implant stability and treatment quality for individual patients. However, despite the application of our workflow, we still observed implant variations, as for instance in regions close to the chest wall (Fig. 5). In this respect, it has to be noted that alterations of the breast tissue itself (e.g., breast swelling/shrinkage) or even slightly different patient breathing phases can influence the in-situ catheter arrangement as well, as mentioned above. In addition to catheter path variations originating from differences in patient positioning, particularly considering various patient breathing phases will have an effect on the dose delivered to the ribs or the chest wall, as described previously [6]. The effect on the dose delivered to further OARs as, e.g., the heart is considered to be much smaller, due to it being located even further away from the implant and the received doses being generally low [18, 19]. However, the exact impact of patient breathing and breast tissue alterations during the treatment course are until now not understood in detail, and will have to be investigated to improve breast brachytherapy further and particularly to be able to individualize treatments patient-specifically.

## Conclusion

Breast positioning control using a vacuum cushion and catheter markings enabled a reduction particularly of larger geometric and dosimetric implant variations for individual patients. This avoided additional effort for treatment re-planning, and led to improved physical plan stability.

### Supplementary Information

Decision-tree analysis of both patient cohorts as well as visualization of geometric and dosimetric implant variations.
