# Supplementary material for: Investigating the impact of breast positioning control on physical treatment parameters in multi-catheter breast brachytherapy
Source: Strahlenther Onkol. 2023 Aug 17;200(1):49–59. doi: 10.1007/s00066-023-02127-0 (PMC10784386; doi:10.1007/s00066-023-02127-0)
Supplement: Supplementary file 1 — Decision-tree analysis of both patient cohorts as well as visualization of geometric and dosimetric implant variations. [file 66_2023_2127_MOESM1_ESM.pdf]

# Supplementary Material A

The following decision-tree, that was published by Karius et al. (reference 6 of the manuscript), was used in clinical routine to identify patients that require treatment adaption by treatment re-planning. The exact methodology of this workflow, which slightly differs from our analysis performed in the present work, can be found and is described in detail in the aforementioned publication. At this place, we present the patient fractions that required further considerations according to the individual tree-steps when treated with or without breast positioning control. The latter cohort is equivalent to the cohort evaluated by Karius et al. in reference 6. This analysis highlighted, as described in the manuscript text of the present work, that our new breast positioning control workflow improves implant stability and hence treatment quality, since no patient required re-planning anymore. From the previous cohort, in comparison, 14% and 1% of the patients required re-consideration due to skin dose variations and target coverage decrease, respectively.

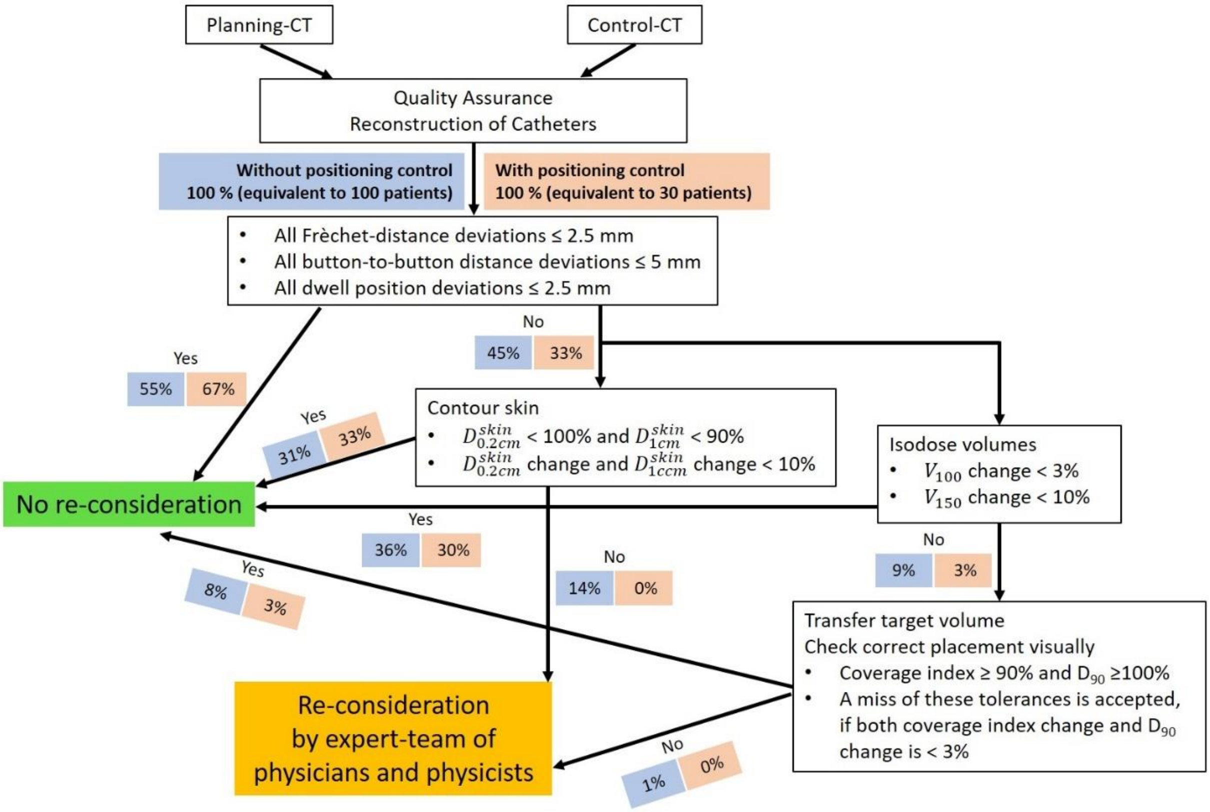


***Fig. SM1:*** *Results of applying our decision-tree methodology to both considered patient cohorts.*

## Supplementary Material B

Shown are the catheter paths (blue) as well as source positions (red) for the two patients of the cohort treated without and with our new workflow, respectively, that showed the maximum geometric implant alterations. Dosimetric results are shown as well, with the 70% (green), 100% (red), 120% (yellow), 150% (cyan), and 200% (dark-blue) isodose lines being illustrated. The left image sections always refer to the planning-CT situation and the right image sections to the control-CT situation, respectively.

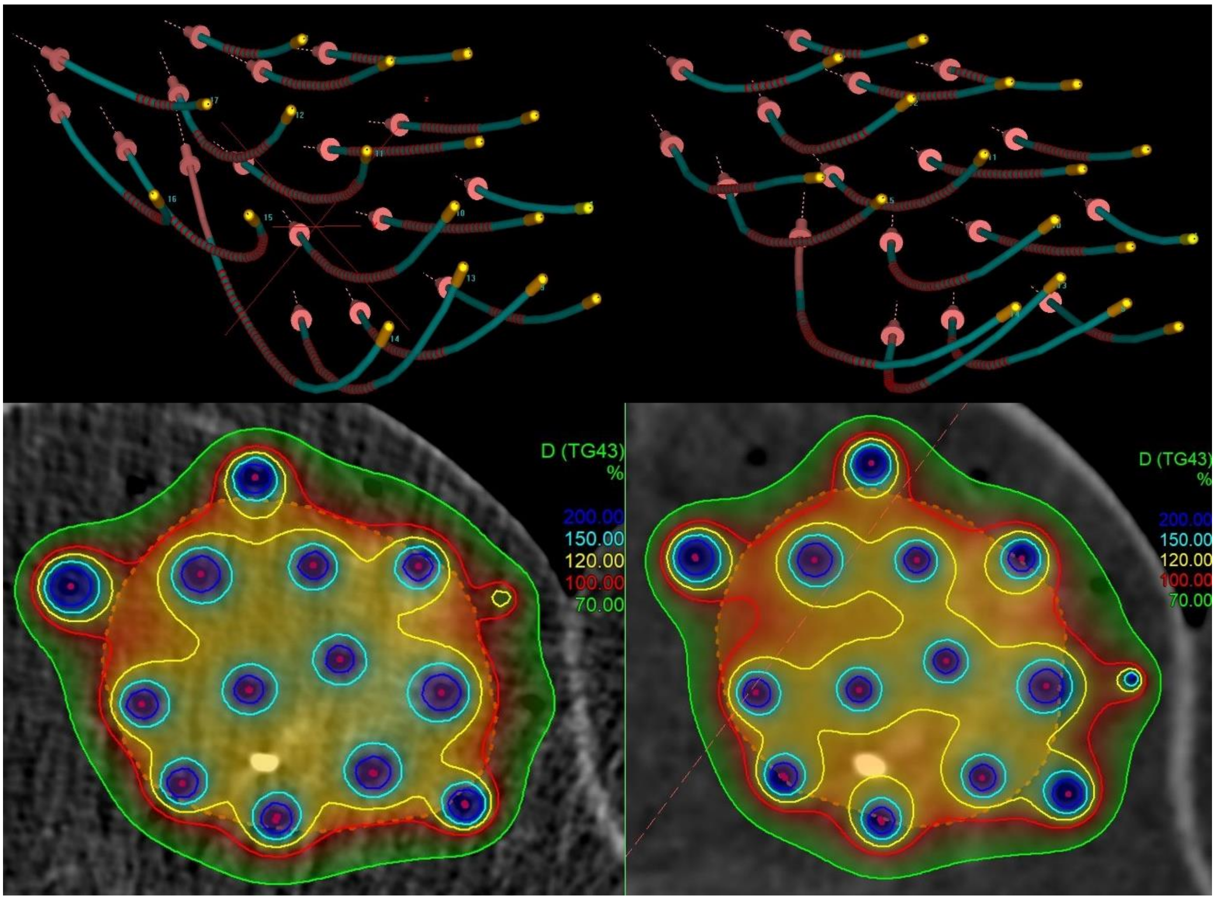


***Fig. SM2:*** *Compared are planning-CT and control-CT situation of the patient showing the maximum dwell position deviation of 14.5mm treated without breast positioning control. The catheter paths were partly completely twisted or shifted, resulting in dosimetric decreases within the target volume (e.g., the 120% isodose volume was much smaller in the control-CT than in the planning-CT).*

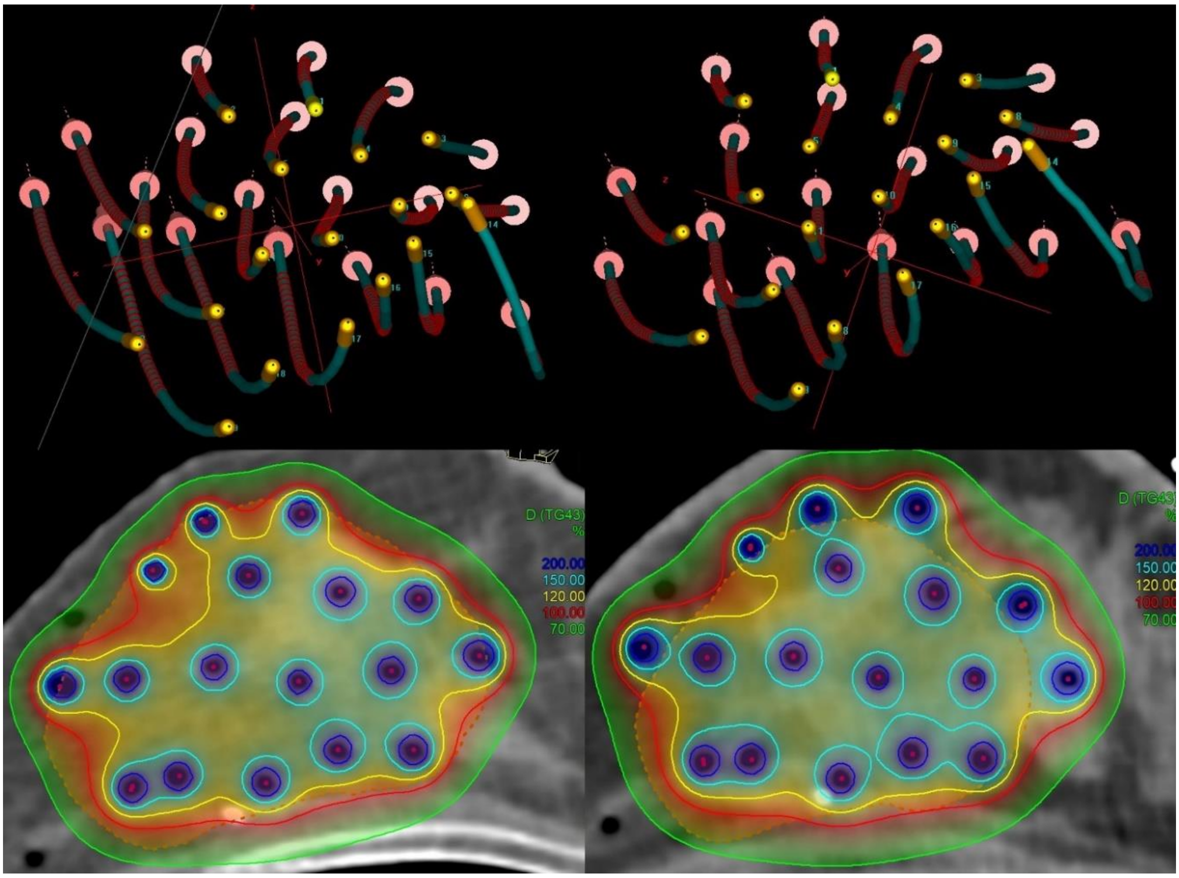


***Fig. SM3:*** *Compared are planning-CT and control-CT situation of the patient showing large dwell position deviations of up to 14mm treated without breast positioning control. The catheter paths have partly clearly changed, resulting in this case in strong increases of the high-dose areas (i.e., the 150% isodose volume was substantially larger in the control-CT than in the planning-CT).*

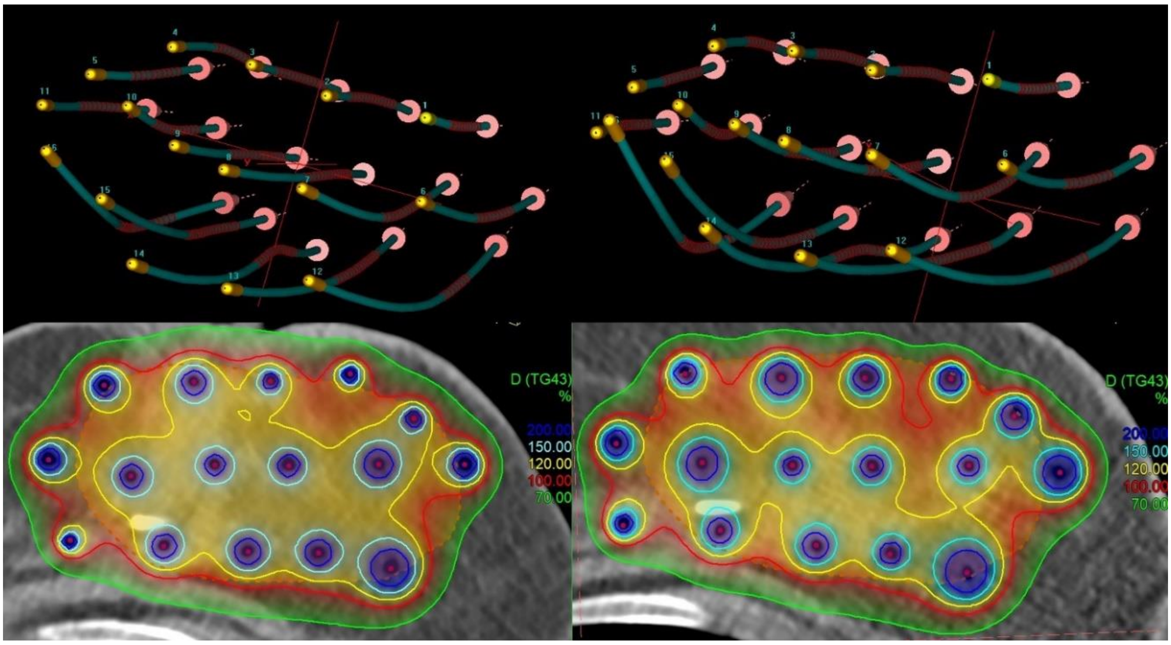


***Fig. SM4:*** *Compared are planning-CT and control-CT situation of the patient showing large dwell position deviations of up to 10mm treated with breast positioning control. Although the implant looked quiet stable, the curvature of individual catheters slightly changed. This resulted in a small reduction in the coverage of the target volume with the prescribed dose.*

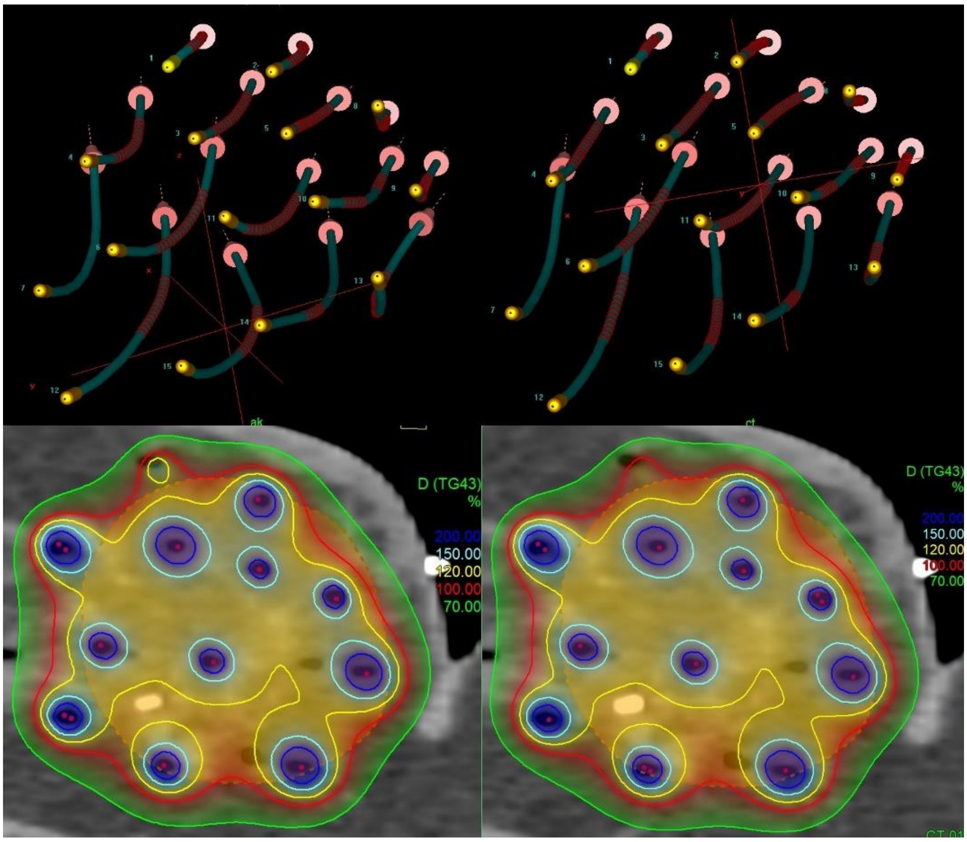


***Fig. SM5:*** *Compared are planning-CT and control-CT situation of the patient showing large dwell position deviations of up to 8mm treated with breast positioning control. Although the implant looked again quiet stable, the curvature of individual catheters particularly in the lowest implant rows changed. However, dosimetric variations were hardly observed in this case.*
